# Fatty-Acid Preference Changes during Development in *Drosophila melanogaster*

**DOI:** 10.1371/journal.pone.0026899

**Published:** 2011-10-27

**Authors:** Anne-Sophie Fougeron, Jean-Pierre Farine, Justin Flaven-Pouchon, Claude Everaerts, Jean-François Ferveur

**Affiliations:** Centre des Sciences du Goût et de l'Alimentation, UMR6265 CNRS, UMR1324 INRA, Université de Bourgogne, Dijon, France; French National Centre for Scientific Research - Université Aix-Marseille, France

## Abstract

Fatty-acids (FAs) are required in the diet of many animals throughout their life. However, the mechanisms involved in the perception of and preferences for dietary saturated and unsaturated FAs (SFAs and UFAs, respectively) remain poorly explored, especially in insects. Using the model species *Drosophila melanogaster*, we measured the responses of wild-type larvae and adults to pure SFAs (14, 16, and 18 carbons) and UFAs (C18 with 1, 2, or 3 double-bonds). Individual and group behavioral tests revealed different preferences in larvae and adults. Larvae preferred UFAs whereas SFAs tended to induce both a strong aversion and a persistent aggregation behavior. Adults generally preferred SFAs, and laid more eggs and had a longer life span when ingesting these substances as compared to UFAs. Our data suggest that insects can discriminate long-chain dietary FAs. The developmental change in preference shown by this species might reflect functional variation in use of FAs or stage-specific nutritional requirements, and may be fundamental for insect use of these major dietary components.

## Introduction

Animal diet is generally shaped to the availability of food resources and to the animal ability to detect and assimilate nutriments. Animals with highly specialized diets are often encountered in poor environments where they have developed peculiar physiological adaptations [1], [2] such as in «plant-insect» or «/parasitoid relationships [3], [4], [5]. In contrast, species with a generalist diet such as *Drosophila melanogaster* often show a much widespread ecological and geographic distribution.

To survive, animals need to ingest essential nutriments that they cannot synthesize. This includes essential fatty-acids (FAs) such as omega-6 FAs in human diet [6], even if their effects have not been yet fully elucidated [7], [8]. However, and despite a strong link between severe dietary based etiologies (obesity, cancer, vascular) and excessive FA consumption, a limited effort was brought to explore the link between the sensory cues provided by FAs and feeding preference [9], [10], [11]. Behavioral studies showed that rodents could discriminate a number of FAs based on their quality and concentration [12], [13], [14]. Insects also show contrasted responses to FAs. For example, the adult mosquitoes *Aedes aegypti* and *Anopheles gambia* and the nymphal bug *Triatoma infestans* are attracted by specific FAs combined with L-lactic acid, which are secreted by the human skin [15], [16], [17]. In contrast, adult mosquitoes and flies may also be repulsed by FAs alone or combined with volatile substances [18], [19].

Since vertebrate and invertebrate organisms show striking similarities in the organization and functioning of their chemosensory systems [20], the fruitfly *D.* is a very useful model to dissect the genetic bases of feeding behaviors. If the biological mechanisms involved in the perception of sugar, salt, bitter substances or amino-acids have been largely explored both in larvae and adults [21], [22], those underlying the perception of FAs received much less attention despite their evolutionary conservation. For example, the CD36 factor required for lipid binding and transport in mammals is also involved in the detection of a FA-derived pheromone in *D. melanogaster* flies [23], [24]. Moreover, sequence similarities exist between members of the ML family of lipid-binding proteins, present in all eukaryotes [25], and the CHEB family of proteins specifically binding to *Drosophila* FA-derived pheromones [26]. However, *Drosophila* response to pure FAs was rarely explored and the few experiments were only done in adults [27].

Using varied behavioral paradigms, involving groups and individuals, we measured the response of wild-type *D. melanogaster* larvae and adults of both sexes to six pure saturated and unsaturated dietary FAs (from C14 to C18). We found that they are able to discriminate these FAs and change their preference during development.

## Materials and Methods

### Strains

In some preliminary tests involving grouped larvae (attraction and repulsion tests) and individual adults (PER), we used two wild-type strains: Canton-S (Cs), a widely used laboratory strain, and Dijon 2000 (Di2 [28]) which was kept in our lab for more than a decade. In the subsequent experiments, we principally used the Di2 strain which showed very robust behavioral performances. Both strains were raised in 150 ml glass vials containing 50 ml of yeast/cornmeal/agar laboratory medium and kept in a breeding room at 24.5±0.5°C with 65±5% humidity on a 12∶12 h light/dark cycle. Strains were transferred every two/three days to avoid larval competition and to regularly provide abundant progeny for testing.

### Chemicals

SFAs (myristic, C14:0; palmitic, C16:0; stearic, C18:0) and *trans*-UFAsoleic, C18:1; linoleic, C18:2; linolenic, C18:3) (Interchim, Montluçon, France). For the larval and PER tests, 10; 100; 200; 500µg were diluted in 50µl of absolute EtOH to obtain the 4 respective concentrations Conc. 0.2, Conc. 2, Conc. 4 and Conc. 10 (given in in µg/µl). For the sake of clarity, we only show the results obtained with the three highest concentrations corresponding to the respective concentrations.

### Larval Behavior

All experiments were performed with the same experimental conditions as described for strains raising. Only early third-instar larvae were used in our tests. To obtain larvae of a synchronized age, they were collected from vials where gravid females had been transferred every two hours. Since FAs cannot be mixed with the hydrophilic buffer used for classical larval tests, a determined amount of pure FA, diluted in 50 µl of absolute ethanol (EtOH), was spotted on a circular zone representing 10% of the surface of the filter paper (see above; Whatman Ashless n°42) which cover all the floor of the observation chamber (9 cm Petri-dish covered with a transparent lid). A few minutes after spotting the FA (and EtOH evaporation), the filter paper was humidified with 1 ml of distilled water. At the beginning of the experiment, ten larvae were simultaneously placed in a neutral area (10% of the surface of the whole filter paper) representing the departure zone at the opposite side of the target zones.

#### Group tests

**Figure 1:**
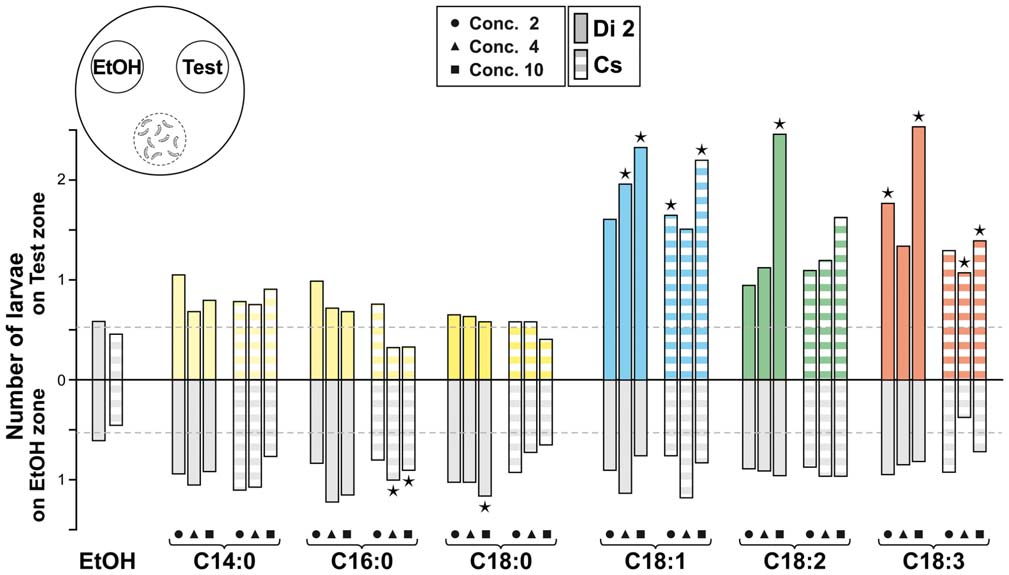
Attraction to fatty-acids in groups of wild-type larvae. Tests involved groups of ten larvae of two wild-type strains: Dijon2000 (Di2; plain) and Canton-S (Cs; striped) placed in the departure zone (pictured in the upper-left cartoon). Their number in each target zone (Test/EtOH) was scored every 5 min. Bars represent the average number of larvae found on the FA zone (Test zone, above base line) and on the control zone (EtOH zone; below the base line) during a 30 min observation period. For each fatty-acid (indicated below the histograms), and for each strain, the three bars correspond to the 3 concentrations used (from left to right: circle  =  Conc. 2, triangle  =  Conc. 4 and square  =  Conc. 10). Stars beneath the bars indicate statistical difference (Chi-square test with a computation of significance by cell) with the control experiment (Test zone  =  EtOH; see the two histograms on the left) represented by the dotted lines below and under the base line (±0.59). N  = 10–12 for each condition.

For the attraction tests, larvae disposed on the departure zone had the choice between two target zones (each one  = 10% of the total area) either covered with 50µl EtOH (solvent control) or with the tested FA (diluted in EtOH; Figure 1). Control experiments included the two target zones similarly covered with EtOH. Experiments lasted 30 min and the number of larvae present on each zone relatively to their total number was noted every 5 min.

For the repulsion tests, ten larvae were simultaneously disposed at the center of a zone covered with FA. The number of larvae moving out of this zone was noted every 30 sec. To measure the aggregative effect induced by FAs, the number of inter-individual contacts (involving ≥3 larvae) was noted every 5 sec. We estimated the “contact index” parameter (CTI) as the summed number of larvae establishing inter-individual contacts during each 5-sec period. The experiment lasted 10 min or less (it was interrupted when the total number of larvae was <3). Some of these experiments were digitally recorded during 10 min with a picture taken every 5 sec (Canon © EOS 1000D, EF-S 18–55).

#### Individual tests

The behavior of single larvae was also measured in control and experimental situations similar to those used in group attraction and repulsion tests. For the attraction test, larval individual behavior was also digitally recorded during 10 min with a picture taken every 5 sec. We measured the locomotor activity, the time latency to reach the FA zone (or the control zone), the time duration on each zone, and the time to re-visit the zone after its first exit (second visit). The locomotor activity, the latency to reach each zone and the time spent on each zone were calculated relatively to the control situation (without FA). We also noted the proportion of first and second visits on each target zone. For the repulsion test, we measured the time taken by each larva to exit out of the test zone.

### Adult behavior

#### Lethality on FA-enriched food

FA-rich food was prepared with 5, 50 or 500 mg FA diluted in 500µL of EtOH with 100 mL warmed-up standard media (to yield the respective concentrations: Conc. 0.05, Conc. 0.5 and Conc. 5). A similar volume of EtOH was added to the control media. The vials or Petri dishes were filled with 3 mL of FA-rich food and used within one week. Thirty one-day-old flies (10 females and 20 males) were introduced into a vial containing FA-rich food and were transferred into fresh FA-rich food vials every 2 days. The number of living flies was assessed at each transfer.

#### Egg-laying behaviour

After mass-mating, five 4- to 5-days old female flies were immediately introduced into a glass container (Duran, 95 mm diameter, 100 mm high with a transparent lid) containing two Petri dishes (Greiner bio-one, 35 mm diameter, 10 mm high) either filled with FA-rich food or with control food. After, 20 hours, the number of eggs was determined on each egg-laying area and the preference index calculated. While transferring groups of adults every 2 days on fresh food to measure their lethality (see above), we also noted the number of eggs with regard to the number of surviving females at each transfer. The total number of eggs was summed for the first 22 days of adult life.

#### Proboscis extension reflex

The proboscis extension reflex (PER) test allowed us to measure the appetence of FAs on individual flies using a standardized procedure [29]. Flies were starved for 20 h before the experiment. One fore-tarsus of a fly was stimulated with a 100 mM sucrose solution (to elicit PER) and the contra lateral fore-tarsus was immediately touched with a small piece of filter paper (2×3 mm) impregnated with FA at Conc. 10.

### Statistics

#### Larval group tests

The larval attraction to different FA concentrations was estimated with a Chi-square test (with a computation of significance by cell) based on the difference between the number of larvae present on the two target zones. With 10 larvae, the random presence of larvae an area representing 10% of the total test area is theoritically “1”. Since the observed values for Di2 and Cs strains were equal to 0.68 and 0.50, respectively, we kept the mean value (0.59) as the control value. Consequently, higher or lower values should respectively indicate an attractive or a repulsive effect of the tested compound.

The repulsive effect of FAs was determined based on the median number of larvae moving out of the zone. The difference between various conditions was tested with a Kruskal-Wallis test completed by Conover & Iman multiple pairwise comparisons (two-tailed with Bonferroni correction). We used the same statistical procedure for the contact index (CTI).

#### Larval individual tests

The response of each larva was analyzed using (i) a Kruskal-Wallis test (with Conover & Iman multiple comparison) for the relative locomotor activity, the relative time spent on each zone and the time to exit out of the test zone, and (ii) a Fisher's exact test to compare the number of first and second visit on each zone.

#### Adult PER inhibition test

For each strain, the frequency of PER inhibition was compared using a Chi-square test (with a computation of significance by cell). All statistical analysis were performed with XLSTAT 2011 software [30].

## Results

We measured the behavioral response of wild-type *D. melanogaster* larvae and adults to the saturated and unsaturated fatty-acids (SFAs: C14:0, C16:0, C18:0; and UFAs: C18:1, C18:2, C18:3, respectively) mentioned in Material and Methods. The preliminary tests were carried out with two wild-type strains (Di2 and Cs) and the more precise tests only with Di2 strain.

### Larval attraction to unsaturated FAs

We first measured the response of grouped larvae to three SFAs three UFAs at three concentrations (Conc. 2, Conc. 4 and Conc. 10; given in µg/µl; see Methods).

The two wild-type larvae clearly preferred UFAs than SFAs (Figure 1). They spent more time on UFAs (average number: 1 to 2.5; Khi2(35 df) = 263.5; p<0.0001) than on SFAs or on the control zone (EtOH; 0.3 – 1; p  =  ns; the dotted value “0.59” corresponds to the random presence of larvae; see Methods). Moreover, larvae (and specially Di2 larvae) showed a dose-dependent response UFAs whereas their frequency did not change on SFAs. The time spent on the control zone was rarely affected by the FA concurrently presented.

**Figure 2:**
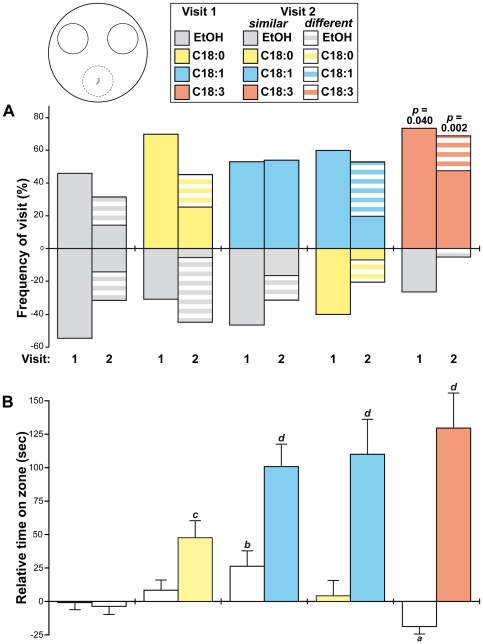
Attraction to fatty-acids in individual wild-type larvae. Individual Di2 larvae were given the choice between two target zones (see cartoon): the frequency of their first and second visits (A; 1 and 2 shown below bars) and the time spend on either zone (B) were measured. (A) The frequency of first visit was calculated from the total number of larvae visiting either zone. Therefore, the sum of the two bars (above + below the base line  = 100%) represents the total number of larvae visiting both zones. The frequency of second visit for larvae visiting a similar zone (plain bars) or a different zone (striped bars) is shown relatively to the zone of their first visit. In this case, the total of the two bars may be <100% since not all larvae showed a second visit. (B) The relative time corresponds to the increased or decreased duration spent in each zone (above or below the base line, respectively) relatively to the control situation (corresponding to the EtOH/EtOH choice: see empty bars on the left). The locomotor activity of individual larvae was also measured (Figure S1). The different letters beneath the bars indicate the statistical difference. (A: Fisher's exact test; B: Kruskal-Wallis test completed by Conover & Iman multiple pairwise comparisons). N  = 15–20.

We investigated more precisely the response of individual Di2 larvae to FAs (Conc. 10). Single larvae showed no preference when they had the choice between EtOH, C18:0 and/or C18:1 (Fisher exact test; p  =  ns). However, in the case of “C18:3/ EtOH” choice, larvae directed their first visit more often to C18:3 than to EtOH (p  = 0.04; Figure 2A). If larvae always spent more time on the FA zone than on the control zone during the first visit, this duration increased on UFAs compared to C18:0 (KW(11 df) = 76.2; p <0.0001; Figure 2B). Moreover, a majority of larvae visiting C18:3 preferentially returned on the zone impregnated with this FA (second visit). Larval locomotor activity strongly decreased with C18:3 and —to a much smaller extent — with EtOH (Figure S1).

### Larval repulsion against saturated FAs

**Figure 3:**
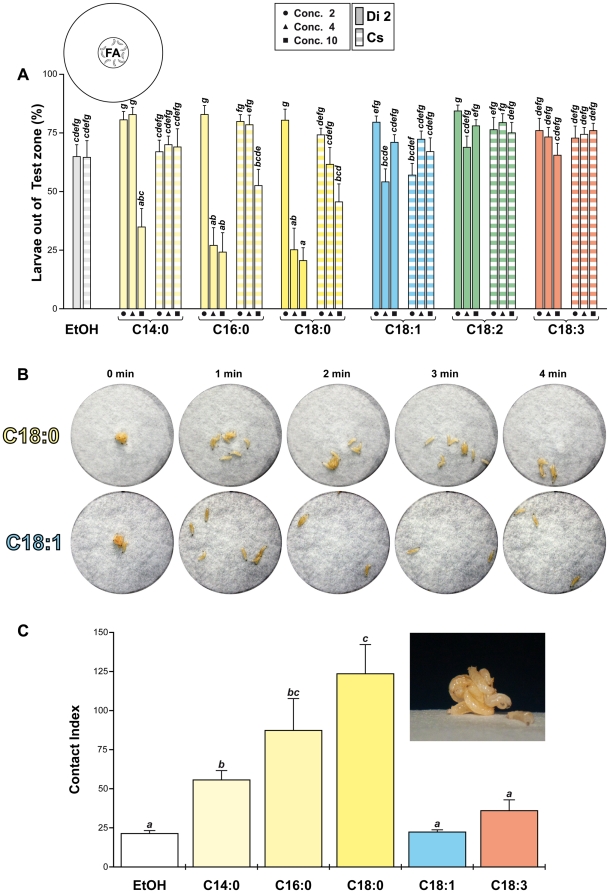
Repulsion against fatty-acids in groups of wild-type larvae. Tests involved groups of ten larvae of two wild-type strains: Dijon2000 (Di2; plain) and Canton-S (Cs; striped) directly placed in the FA zone (see cartoon). Each test lasted 10 min, and the number of larvae out of the FA zone was noted every 30 seconds (See Figure S2). (A) Bars represent the median number of larvae out of the FA zone. For each fatty-acid (indicated below the histogram), and for each strain, the three bars correspond to the 3 concentrations used (from left to right: circle  =  Conc. 2, triangle  =  Conc. 4 and square  =  Conc. 10). N  = 10. (B) A picture was taken every min (until 4 min) to show the larval dispersion on C18:0 (top) and C18:1 (bottom; see the movies shown in the supplemental information). (C) The number of inter-individual contacts (involving ≥3 larvae), noted every 5 sec, was used to calculate the “contact index” (CTI; see Material and methods). The upper-right picture shows a typical aggregation cluster of larvae placed on C18:0. N  = 20. For B and C, we used the Conc. 10. The different letters shown above the bars indicate the significant differences. All tests (A and C) were performed with Kruskal-Wallis test completed by Conover & Iman multiple pairwise comparisons. The repulsive effect of FAs was also measured on the behavior of single larvae (Figure S3).

To measure the repulsive effect potentially induced by FAs, we noted the rapidity for 10 larvae (Di2 or Cs) to exit the FA zone (Figure S2). Unexpectedly, their exit on SFAs was delayed comparatively to UFAs (Figure 3A; KW(49 df) = 180.4; p <0.0001). This effect was particularly persistent (>10 min) with Di2 larvae tested on the higher SFAs concentrations. A closer examination revealed that, at the contact of SFAs, larvae established more frequent inter-individual contacts than on UFAs, and this may explain their delayed exit out of SFAs (Figure 3B; see Movie S1 for EtOH, Movie S2 for C18:0 and Movie S3 for C18:1). More precisely, the frequency of inter-individual contacts («contact index» CTI; see Methods) significantly increased with the chain length of SFAs (KW(5 df) = 64.5; p <0.0001; Figure 3C) whereas it was not altered on EtOH and UFAs.

The behavior of single individual larvae was also measured: their time to exit out of the FA zone strongly increased on SFAs compared to EtOH and UFAs (KW(5 df) = 56.5; p <0.0001; Figure S3). This pattern was very similar to the “Contact Index” shown by grouped larvae on respective FAs (Figure 3C).

### Adult individual appetence for FAs

**Figure 4:**
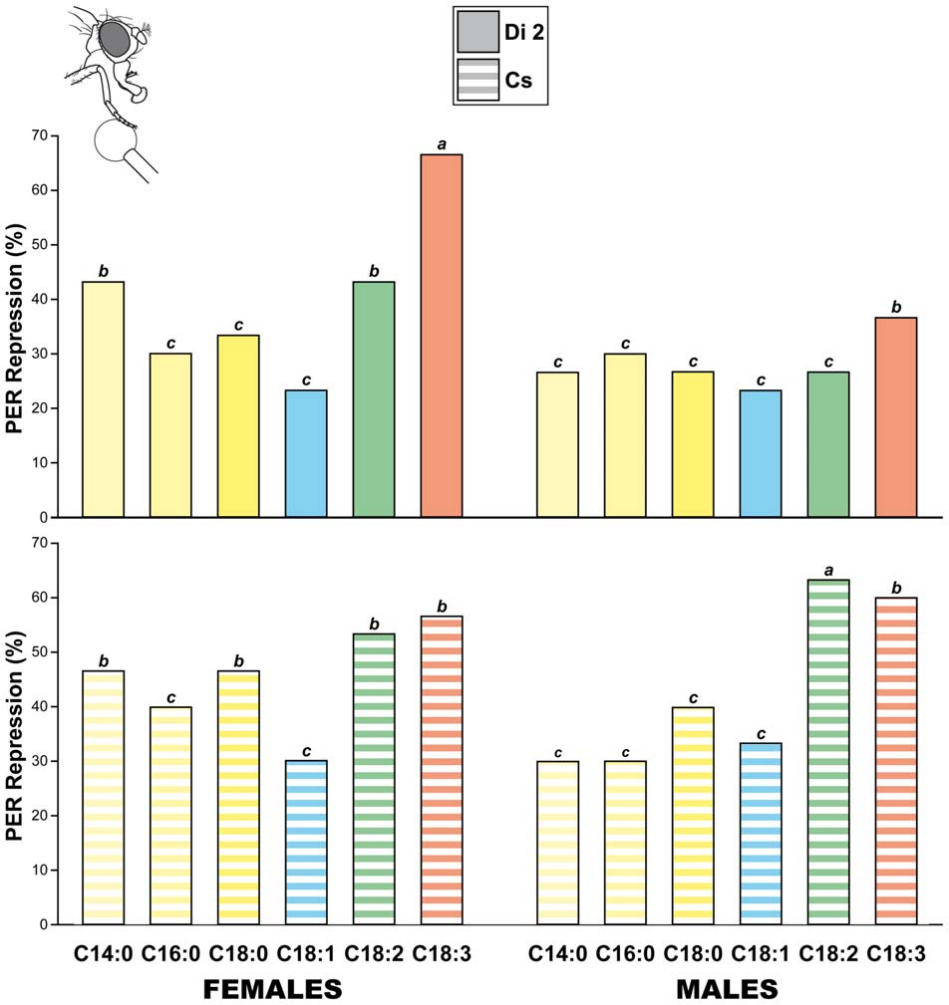
Repression of appetitive behavior by fatty-acids in individual adults. Tests involved individual adults of two wild-type strains: Dijon2000 (Di2; plain color; top) and Canton-S (Cs; striped color; bottom). Bars indicate the repression average of individual proboscis extension reflex (PER; see cartoon) indexes in response to six FAs (shown below) at Conc. 10. Flies who extended their proboscis and opened their labellum were counted as PER positive flies. The repression of the PER index corresponds to the frequency of individual flies, initially stimulated by the unilateral stimulation by sucrose, whose response was repressed after the bilateral application of the FA. The different letters shown above the bars indicate the significant differences for each strain (Chi-square test with a computation of significance by cell). N  = 30 for each FA condition, strain, and sex.

Since food preference can change with aging, we also investigated the adult responses to FAs. First, we measured the proboscis extension reflex (PER) in individual Cs and Di2 male and female flies. We estimated the ability of FAs to repress PER in flies initially stimulated with sucrose the difference between (*i*) the number of PER induced on flies unilaterally stimulated by sucrose minus (*ii*) the number of PER on flies bilaterally stimulated by sucrose and FAs (Figure 4).

The tested FAs (at Conc. 10) induced a sex- and strain-specific effect. In Di2 flies, C18:3 had the strongest effect (67% females and 36% males repressed) whereas C14:0 and C18:2 induced a milder effect only in Di2 females (43%; Khi2(11 df) = 22.3; p  = 0.022). In Cs flies, both C18:2 and C18:3 induced the strongest effect (55–65% repressed), whereas C14:0 and C18:0 had a milder effect in Cs females (48%; Khi2(11 df) = 19.8; p  =  0.048). In both strains, the other FAs tested induced a weaker effect (about 30% flies repressed).

### Adult preference and fitness on FAs

To further explore the adult effect of FAs, we measured egg-laying behavior and lethality in groups of Di2 flies kept on food enriched with C18:0, C18:1 or C18:3 (at Conc. 0.05, Conc. 0.5 and Conc. 5).

#### Egg-laying behaviour

**Figure 5:**
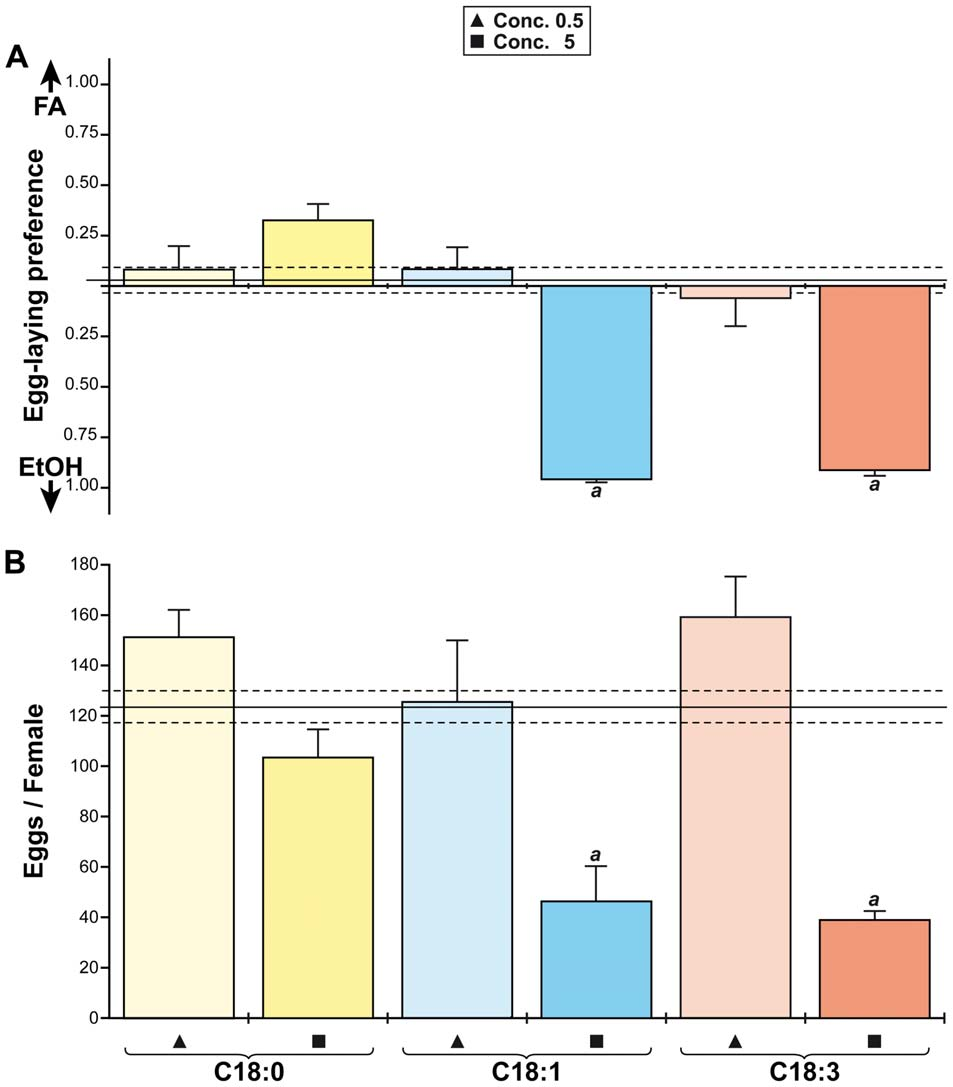
Egg-laying behavior on fatty-acids. Young Di2 females recently mated were given a choice of two types of food (A; mixed with FA, above base-line or with EtOH, below base-line) to lay eggs. The total number of eggs laid on each type of food was noted after 20 hours (Figure S4) and after 20 days (B). For each FA, two concentrations were used: Conc. 0.5 (triangle) and Conc. 5 (square; shown below the histograms). (A) The egg-laying preference, estimated from the difference of eggs found on each type of food, indicate a preference either for the FA (above the base line) or for EtOH (below the base line). Letters indicate a significant effect of the food compared to the control situation (between the two dotted lines; Kruskal-Wallis test completed by Conover & Iman multiple pairwise comparisons). N = 10 and 14–17 for experiments A and B, respectively.

Young mature females were given the choice, during a 20 hours period, to lay eggs on two batches of food enriched with FA (Conc. 0.5, Conc. 5) or EtOH (control). They showed a strong avoidance against the higher UFA concentration whereas the lower UFA concentration and C18:0 had no effect (KW(6 df) = 76.7; p<0.0001; Figure 5A). If the repulsive effect of UFAs did not negatively affect the number of eggs laid during the 20 hour-long experiment (Figure S4), it strongly impacted the total number of eggs laid during the 22 days following mating. Females laid much less eggs (40) on the two UFAs at Conc. 5 than (*i*) at Conc. 0.5 and (*ii*) on C18:0 (100–160 eggs/female; KW(9 df) = 54.2; p<0.0001; Figure 5B).

#### Adult survival

**Figure 6:**
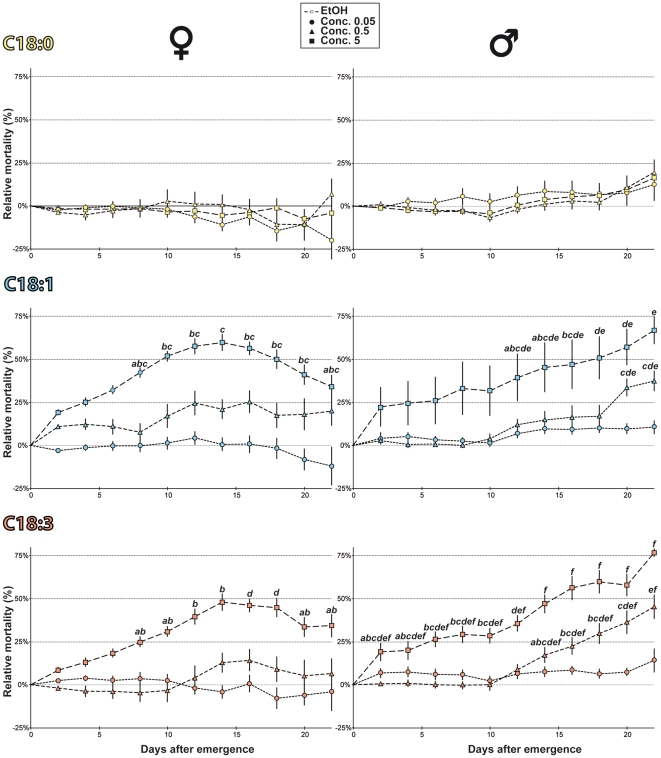
Adult survival on fatty-acids. The mortality induced by FA-rich food was counted every two days and during 22 days of adult life on female and male Di2 adults (left and right, respectively). Graphs are shown relatively to same-sex flies kept on plain food (% relative mortality). For each sex and FA (from top to bottom: C18:0, C18:1, C18:3), we used three concentrations: Conc. 0.05 (circles), Conc. 0.5 (triangles) and Conc. 5 (squares). The different letters indicate the significant differences for each genotype and FA. (Kruskal-Wallis test completed by Conover & Iman multiple pairwise comparisons). N  = 10.

C18:0, C18:1 and C18:3 also very differently affected the survival of adult females and males. The lower FA concentration (Conc. 0.05) had no major effect with regard to the lethal time at which 50% flies had died (LT50: 18.1 to 19 days for females, 17.1 to 23.1 days for males). Higher UFA concentrations induced earlier lethality: Conc. 5 of C18:1 and C18:3 practically halved the LT50 in both sexes, as well as Conc. 0.5 of C18:1 in females (10.4–12.9 days). Conversely, C18:0 had no (or a slight effect) on adult survival in either sex. Our dynamic curves (Figure 6) indicate that both UFA at Conc. 5 affected survival in females earlier (8 day-old) than in males (12 day-old). However, after 14 days, with the two UFAs at Conc. 0.5 and Conc. 5, males showed a more important relative lethality than females.

## Discussion

*Drosophila* larvae and flies showed very contrasted preferences to pure FAs. Larvae clearly preferred UFAs and disliked SFAs whereas adults showed a somewhat reciprocal preference.

### Multiple roles of FAs

The essential role of FAs in the mammal's diet may be due to their critical role in reproduction, cold adaptation and metabolism [6]. In insects, a change of FA content can affect female fecundity [31] and remating [32], as well as their production of sex pheromones [33]. The presence and composition of FAs in cell membranes can also affect cold adaptation in *Drosophila* [34]. Arachidonic (C20:4) and C18:1 acids are involved in *Drosophila* cell signaling by activating transient receptor potential (TRP) channels [35], [36], some of which may be required in taste transduction [37]. The metabolic state may be linked with FA preference and ingestion: human oral hypersensitivity to FAs is associated with lower energy and fat intake [38] whereas *Drosophila* larvae fed with long chain dietary FAs show a reduced tolerance to EtOH [39].

### Multiple sensory perception of FA

Previous rodent and insect studies showed that FAs could induce contrasted behavioral effects according to their nature and concentration. At low concentrations, C18:2 induces an appetitive response in rodents and human whereas, at higher concentrations, it has an unpleasant effect [12], [14], [38]. This effect may be related to the activity of K+ channels in the taste cells of the fungiform papillae which can be inhibited by long-chain polyunsturated FAs but not by SFAs ([40]. Moreover, the textural properties of FAs (smoothness) may be perceived by the trigeminal system of mammals to provide a mechanosensory information complementary to olfactory and gustatory cues provided by FAs [41].

Our experiments suggests that *Drosophila* larvae use distinct sensory systems to perceive FAs. The increased duration spent on UFAs, as well as the decreased locomotor activity on C18:3, indicate the involvement of larval taste. The significantly increased frequency of first and second visits on C18:3 suggests respectively that (i) larvae can smell this FA before taste contact and (ii) like this FA since they returned on it for a second time. Larvae may also detect high concentration of SFAs using mechanosensory organs involved in perception of the «textural» features of FA especially since lipids can activate mechanosensitive channels [42], [43], [44]. Mechanosensory perception, possibly associated with nociceptive perception, may explain why individual larvae spent more time on SFAs compared to UFAs. We particularly noted that individual and grouped larvae placed on SFAs frequently showed non-typical behaviors (frequent turning, roll-over, standing upright) indicating that larvae were stressed. This is the reason why we believe that larvae grouped on SFAs increased their frequency of inter-individual contacts to avoid “touching” high SFA concentrations. However, the similarity of the behavioral patterns shown by individual and grouped larvae (Figure S3 and Figure 3, respectively), with regard to the FAs tested, indicates that individual locomotor behavior was not affected by aggregation behavior. Enhanced aggregation behavior in larvae could reflect a cooperative response to a stressful situation either to increase their protection against a predator or to increase the use of resources ("Allee effect"; [45], [46]). Based on the fact that insect pheromones — some of which are FA-derived — can induce aggregation behavior in adult insects (*Drosophila*, [47]; Cerambycid beetles, [48]), and in their larvae (Potato beetle, [49]; *Triatomid* bugs, [50]; Codling moth, [51]), we postulate that *D. melanogaster* larvae emit aggregation pheromones in response to the stress induced by SFAs. In adult insect, the emission of aggregation pheromones in response of a food source was mainly described in bark beetle species of the two genera *Ips* and *Dendroctomus* [52]. Moreover, *D. melanogaster* adults are know to aggregate in response to the effect of a male pheromone (*cis*-vaccenyl acetate) combined with some volatile food compounds [53].

If the results obtained in the attraction test and in the repulsion test are not reciprocal, this apparent discrepancy may be related to our experimental design: in the attraction test, larvae are free to visit or to avoid the FA zone whereas in the repulsion test, larvae have no choice. In the latter case, the stress possibly induced by SFAs seems to disorganize the larval pathway which is chaotic compared to the pattern shown by larvae on UFAs.

### Developmental change for FA preference

Our results clearly show that the FA preference of adults is changed compared to larvae: the higher concentrations of C18:1 and C18:3 induced a more negative effect — on PER repression, egg-laying behavior and lethality — than C18:0. In comparison, lower UFA concentrations had no or a lesser effect on both adult phenotypes. If the two wild-type strains showed a somewhat similar contrasted PER pattern to SFAs *vs* UFAs, Di2 flies showed an enhanced sexual dimorphism compared to Cs flies. A strain-specific effect in the adult response to FAs was previously reported [27]. Since the two wild-type strains differ for their genetic background, the divergence of their response could result of a gene x environment interaction [54]. FAs also induced sex-specific effect on adult lethality: males exposed to the highest UFA concentrations died more frequently than females. If Di2 females are less sensitive than Di2 males to the toxic effect of UFAs, the very reduced number of eggs laid on UFA-rich food suggests that females are strongly repulsed by these substances. *Drosophila* females also show an higher ability than males to detect sucrose, bitter substances and a FA-derived sex pheromone [55], [56].

More generally, our experiments reveal a change of FA preference during lifespan. The convergent effects obtained with the three larval experiments, on one side, and those obtained with the three adult experiments, on the other side, allow us to rule out the possibility of an experimental artifact caused by (*i*) the design of the behavioral assays, or (*ii*) the interaction between FAs and other substances (EtOH, food) used in our tests.

Could the preference shift from larva to adult reflects different dietary requirement during *Drosophila* development as shown for different types of sugars [57]? If the effect of essential FAs varies between insects, Lepidoptera and Hymenoptera absolutely need C18:2 and C18:3 to achieve their complete metamorphosis [58]. Conversely, if mosquitoes do not need these FAs to survive, they need these substances to fly [59]. If the nature and effect of essential FAs remain unknown in *Drosophila*, the ingestion of yeast influence the quantity and quality of FA stored [60], [61]. A change of FA preference could also broaden food resources and reduce food competition. A wide physiological adaptation to varied sources of food could partly explain why this generalist species can survive on many food types even if larval exposure to a food source can moderately change adult preference to this type of food [62].

In summary, we found that *Drosophila* show marked FA preference which change during life. Our future aim will consist to identify some of the genes that affect larval and/or adult preference and measure to which extent sensory exposure can change preference.

## Supporting Information

Figure S1 **Locomotor activiy of single larvae in a choice test.** Larvae were given a choice between two zones that were either covered (*i*) with EtOH and a fatty-acid (C18:0, C18:1 or C18:3), or (*ii*) both with EtOH (control) or (*iii*) both with two different FAs (C18:0 *vs*. C18:1; see upper-left cartoon). Bars represent the mean activity (±sem; in mm) shown relatively to the activity of single larvae tested in a similar device without any chemical (151.7±3.4 mm between t = 0–2.5 min; 145.4±5.0 mm between t = 2.5–5.0 min; 147.4±5.4 mm between t = 5.0–7.5 min; 133.1±5.8 mm for t = 7.5–10.0 min). Larvae were tested in conditions similar to those described on Figure 2 with a Kruskal-Wallis test completed by Conover & Iman multiple pairwise comparisons. N = 15–20.
(TIF)

Figure S2 **Dynamic exit out of fatty-acid zone.** The cumulated proportion of larvae moving out the zone covered with a fatty-acid was measured every 30 sec during a total of 10 min (see upper-left cartoon). Six fatty-acids (C14:0, C16:0, C18:0, C18:1, C18:2, C18:3; indicated above each set of curves) were tested at three concentrations (Conc. 0.05 =  circles; Conc. 0.5 =  triangles; and Conc. 5 =  squares). For methods, refer to the text and legend of Figure 3.
(TIF)

Figure S3 **Behavior of individual larvae on fatty-acid.** Single larvae were disposed at the center of a FA zone (a filter paper impregnated with diverse FAs at Conc.10). Bars represent the mean (±sem) time to exit out of the test zone. N  = 15. For statistics and methods, see the legend of Figure 3.
(TIF)

Figure S4 **Egg-laying behavior on fatty-acids after 20** **hours.** Recently mated two-days old Di2 females were given a choice of two types of food (mixed with FA or with EtOH). Three FAs (C18:0, C18:1 and C18:3) were tested at two concentrations (Conc. 0.5 =  triangle; Conc. 5 =  square). Bars represent the mean total number of eggs (±sem) laid during 20 hours on the two zones (FA + EtOH). These data correspond to the results shown on Figure 5A. For methods and statistics, please refer to the legend of Figure 5.
(TIF)

Movie S1 **A group of 10 larvae was disposed in a Petri dish at the center of a zone covered with ethanol (EtOH; movie EtOH).** One picture was taken every 10 sec for a total duration of 10 min.
(m4v)

Movie S2 **A group of 10 larvae was disposed in a Petri dish at the center of a zone covered with stearic acid (C18:0) at the Conc.10 concentration.** One picture was taken every 10 sec for a total duration of 10 min.
(m4v)

Movie S3 **A group of 10 larvae was disposed in a Petri dish at the center of a zone covered with oleic acid (C18:1), at the Conc.10 concentration.** One picture was taken every 10 sec for a total duration of 10 min.
(m4v)
