# Supplementary figures and images for: Fatty-Acid Preference Changes during Development in Drosophila melanogaster
Source: PLoS One. 2011 Oct 27;6(10):e26899. doi: 10.1371/journal.pone.0026899 (PMC3203165; doi:10.1371/journal.pone.0026899)

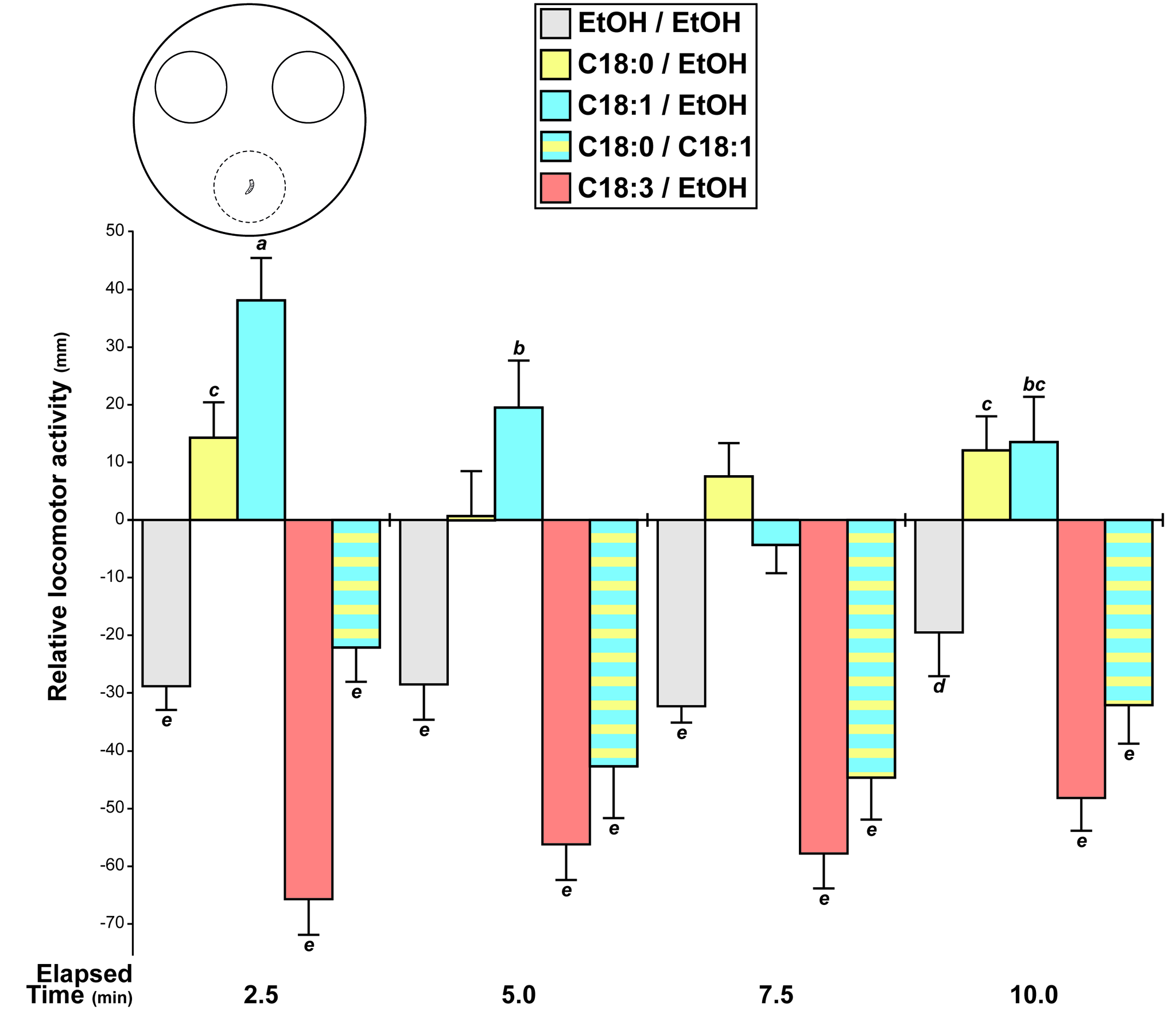

Supplement: Figure S1 — Locomotor activiy of single larvae in a choice test. Larvae were given a choice between two zones that were either covered (i) with EtOH and a fatty-acid (C18:0, C18:1 or C18:3), or (ii) both with EtOH (control) or (iii) both with two different FAs (C18:0 vs. C18:1; see upper-left cartoon). Bars represent the mean activity (±sem; in mm) shown relatively to the activity of single larvae tested in a similar device without any chemical (151.7±3.4 mm between t = 0–2.5 min; 145.4±5.0 mm between t = 2.5–5.0 min; 147.4±5.4 mm between t = 5.0–7.5 min; 133.1±5.8 mm for t = 7.5–10.0 min). Larvae were tested in conditions similar to those described on Figure 2 with a Kruskal-Wallis test completed by Conover & Iman multiple pairwise comparisons. N = 15–20. (TIF) [file pone.0026899.s001.tif]

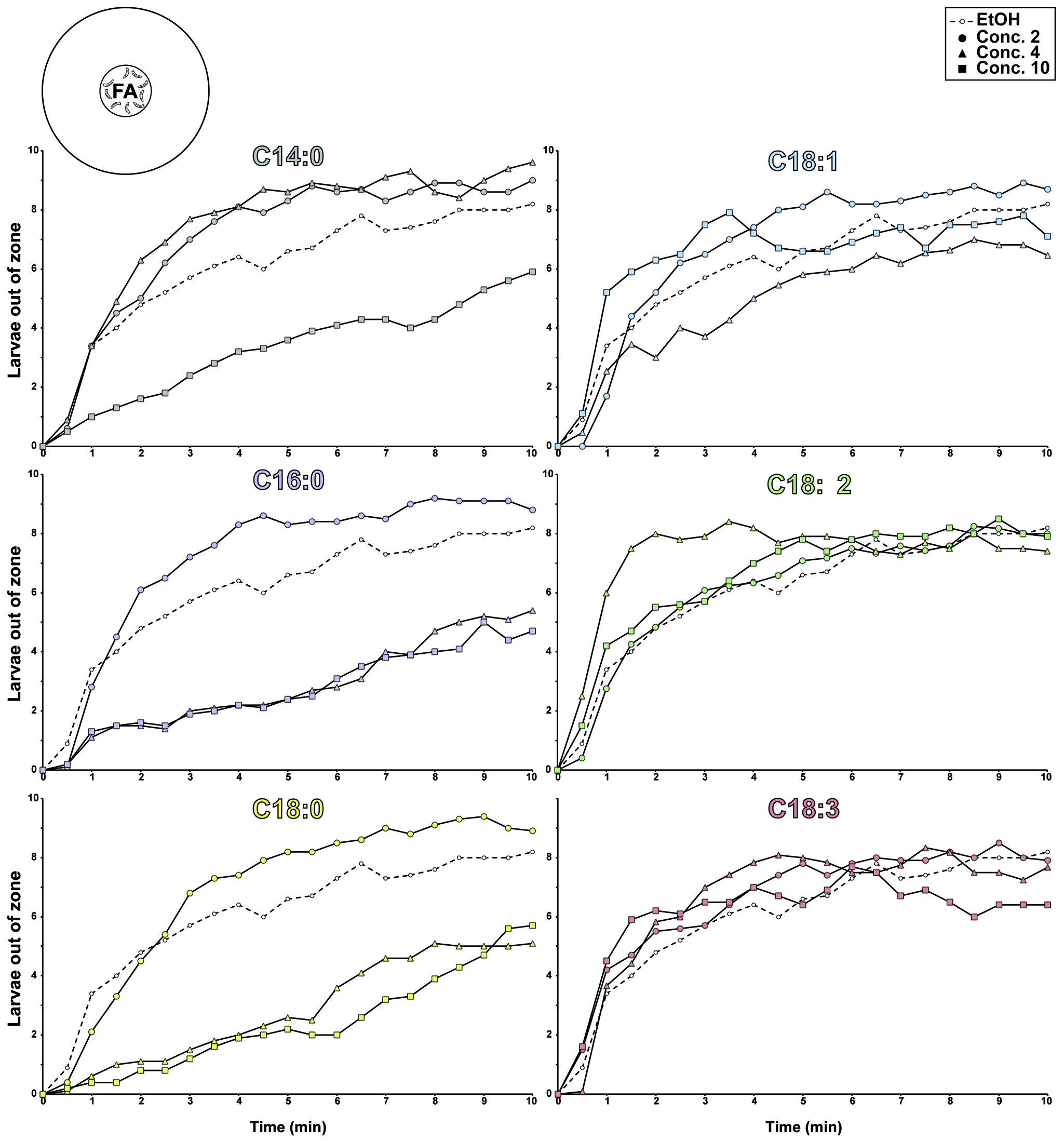

Supplement: Figure S2 — Dynamic exit out of fatty-acid zone. The cumulated proportion of larvae moving out the zone covered with a fatty-acid was measured every 30 sec during a total of 10 min (see upper-left cartoon). Six fatty-acids (C14:0, C16:0, C18:0, C18:1, C18:2, C18:3; indicated above each set of curves) were tested at three concentrations (Conc. 0.05 = circles; Conc. 0.5 = triangles; and Conc. 5 = squares). For methods, refer to the text and legend of Figure 3. (TIF) [file pone.0026899.s002.tif]

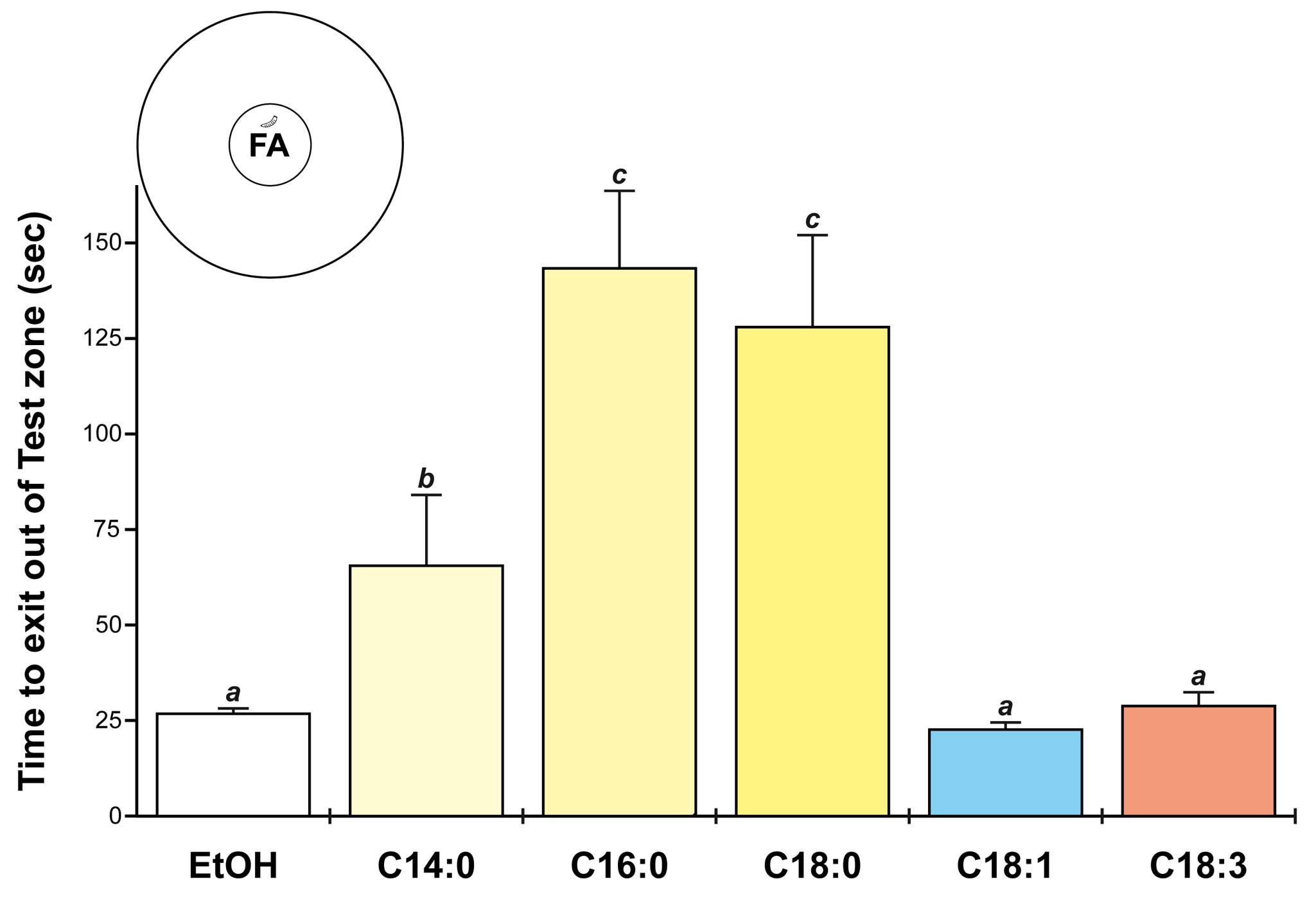

Supplement: Figure S3 — Behavior of individual larvae on fatty-acid. Single larvae were disposed at the center of a FA zone (a filter paper impregnated with diverse FAs at Conc.10). Bars represent the mean (±sem) time to exit out of the test zone. N = 15. For statistics and methods, see the legend of Figure 3. (TIF) [file pone.0026899.s003.tif]

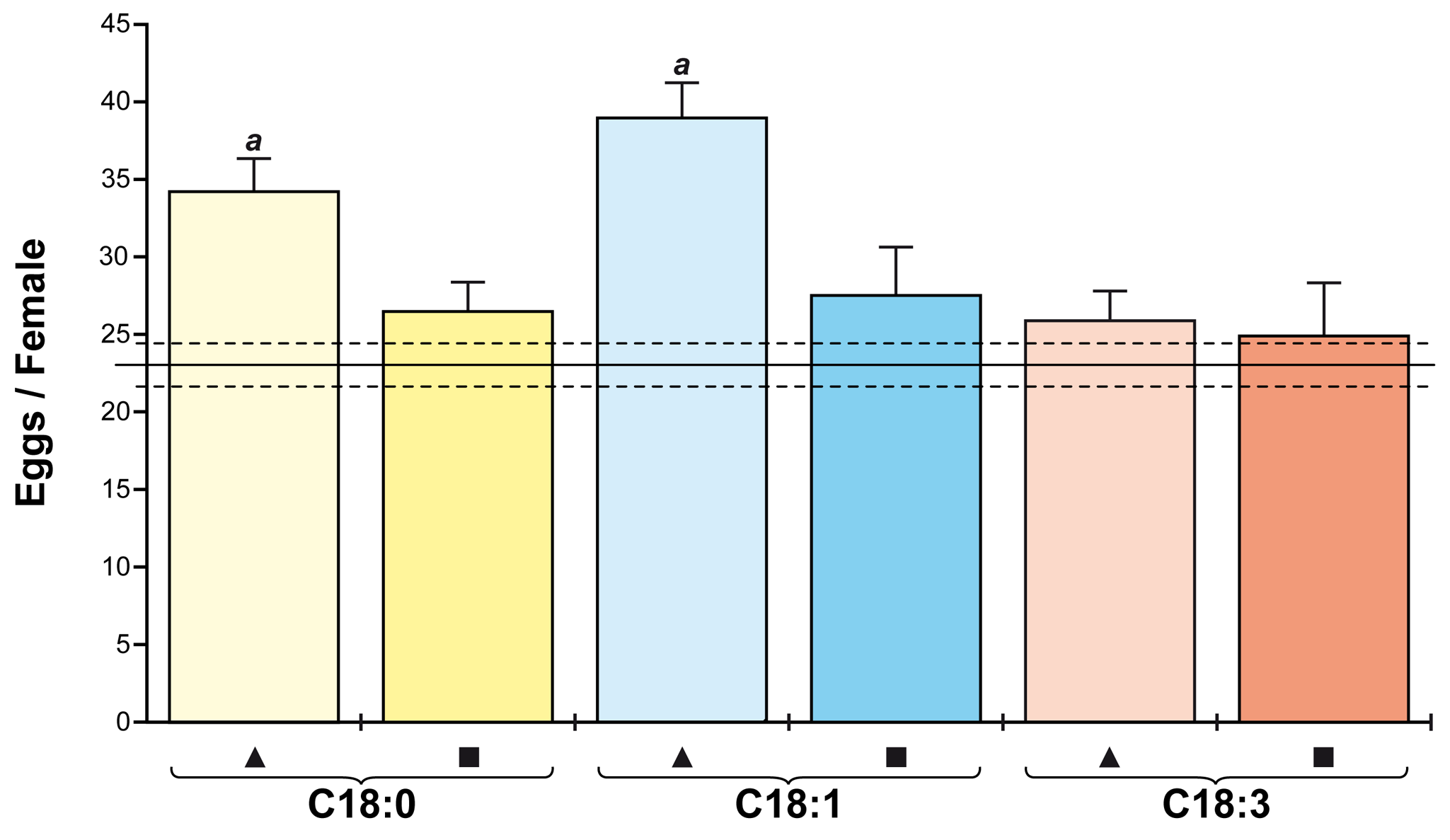

Supplement: Figure S4 — Egg-laying behavior on fatty-acids after 20 hours. Recently mated two-days old Di2 females were given a choice of two types of food (mixed with FA or with EtOH). Three FAs (C18:0, C18:1 and C18:3) were tested at two concentrations (Conc. 0.5 = triangle; Conc. 5 = square). Bars represent the mean total number of eggs (±sem) laid during 20 hours on the two zones (FA + EtOH). These data correspond to the results shown on Figure 5A. For methods and statistics, please refer to the legend of Figure 5. (TIF) [file pone.0026899.s004.tif]
